# The complete chloroplast genome of *Pluchea pteropoda* Hemsl, a mangrove associate plant

**DOI:** 10.1080/23802359.2021.1930600

**Published:** 2021-05-24

**Authors:** Zhenbiao Liang, Xuena Xie, Yongshan Liang, Haiyong Zhang, Weiguo Zhao, Lei Tang, Zhi Chao

**Affiliations:** a Department of Pharmacy, Zhongshan Municipal People’s Hospital, Zhongshan, China; b Faculty of Medicinal Plants and Pharmacognosy, School of Traditional Chinese Medicine, Southern Medical University, Guangzhou, China

**Keywords:** Chloroplast genome, phylogenomic analysis, *Pluchea pteropoda*

## Abstract

*Pluchea pteropoda* Hemsl is a mangrove associate plant of Asteraceae with medicinal properties such as anti-inflammation and fever-relieving. Here, our study presented the complete chloroplast (cp) genome of *Pluchea pteropoda* Hemsl. The cp genome of *P. pteropoda* was 152,300 bp in length, including a large single copy (LSC) region of 84,127 bp, a small single copy (SSC) region of 18,093 bp and a pair of inverted repeats (IR) regions of 25,040 bp. A total of 111 unique genes were found, comprising 79 protein-coding genes, 28 tRNA genes, and 4 rRNA genes. The GC content of the cp genome was 37.5%. Phylogenetic analysis suggested that *P. pteropoda* nested in *Pluchea* clade, which was closely related to *Ageratina adenophora* and *Senecio scandens*. The work provides beneficial data for following researches on the genetic variation, species delimitation, phylogeny and classification of *Pluchea* genus.

*Pluchea pteropoda* Hemsl is a mangrove associates’ plant in the Asteraceae family. The species inhabits land-based sandy beaches, rock crevices or places where high tide can reach, along the coasts of South China and the Indochina peninsula (Linh et al. 2013). Many plants of the genus *Pluchea* have antioxidant, anti-inflammatory and neuropharmacological effects (Thongpraditchote et al. 1996; Sen et al. 2002; Barros et al. 2006). It has been found that methanol extracts from barks and leaves of *P. pteropoda* showed certain antibacterial activity (Yuliani et al. 2015). In Vietnam, *P. pteropoda* has long been used as a folk medicinal herb, often used to treat fevers (Luger et al. 2000). So far, only the cp genome of *P. indica* has been reported in the genus *Pluchea*. Herein, we reported the cp genome of *P. pteropoda*, in order to provide significant information for its further studies.

The materials of *P. pteropoda* were collected from Xinzhou Town, Danzhou City, Hainan Province, China (109°17′33.43″E, 19°42′11.48″N). The voucher specimen (Chao Zhi 20200405016) was identified by Professor Zhi Chao and deposited in the herbarium of the School of Traditional Chinese Medicine, Southern Medical University (contact Zhi Chao, chaozhi@smu.edu.cn). Total genomic DNA was extracted from 100 mg fresh leaves using cetyltrimethylammonium bromide (CTAB) method (Yang et al. 2014). The Pair-end (PE) sequencing was performed on BGISEQ500 system at the Beijing Genomics Institution (BGI), Shenzhen, China. The cp genome of *P. pteropoda* was assembled by SPAdes (Bankevich et al. 2012) with *Pluchea indica* reference (Accession No. NC038194). The Geneious (v20.0.4) (Drummond et al. 2012) and Plastid Genome Annotator (PGA) (Qu et al. 2019) were used for genome annotation. The annotated sequence had been deposited in GenBank (Accession No. MW554520).

The complete cp of *P. pteropoda* displayed a typical quadripartite structure with 152,300 bp in length, including a large single copy (LSC) region of 84,127 bp, a small single copy (SSC) region of 18,093 bp and a pair of inverted repeats (IR) regions of 25,040 bp. A total of 111 unique genes were annotated, consisting of 79 protein-coding genes, 28 tRNA genes, and 4 rRNA genes. Eighteen genes containing intron were found. Among them, fifteen genes contained one intron and three genes (*clpP, rps12, ycf3*) contained two introns. The overall GC content of the chloroplast genome was 37.5%.

A phylogenetic analysis was performed based on 14 cp genomes to reveal the phylogenetic position of *Pluchea pteropoda*. The cp genomes were aligned with MAFFT (Katoh and Standley 2013) and then adjusted manually by Mega7 (Kumar et al. 2016). The GTR + F+G4 was selected as best-fit model of DNA evolution by ModelFinder (Kalyaanamoorthy et al. 2017). Phylogenetic tree was constructed using RAxML version 8.2.10 (Stamatakis 2014) with 1000 bootstrap replicates. Ultimately, the phylogenetic analysis result showed that *P. pteropoda* nested in *Pluchea* clade and was closely related to *Ageratina adenophora* and *Senecio scandens* (Figure 1).

**Figure 1.**
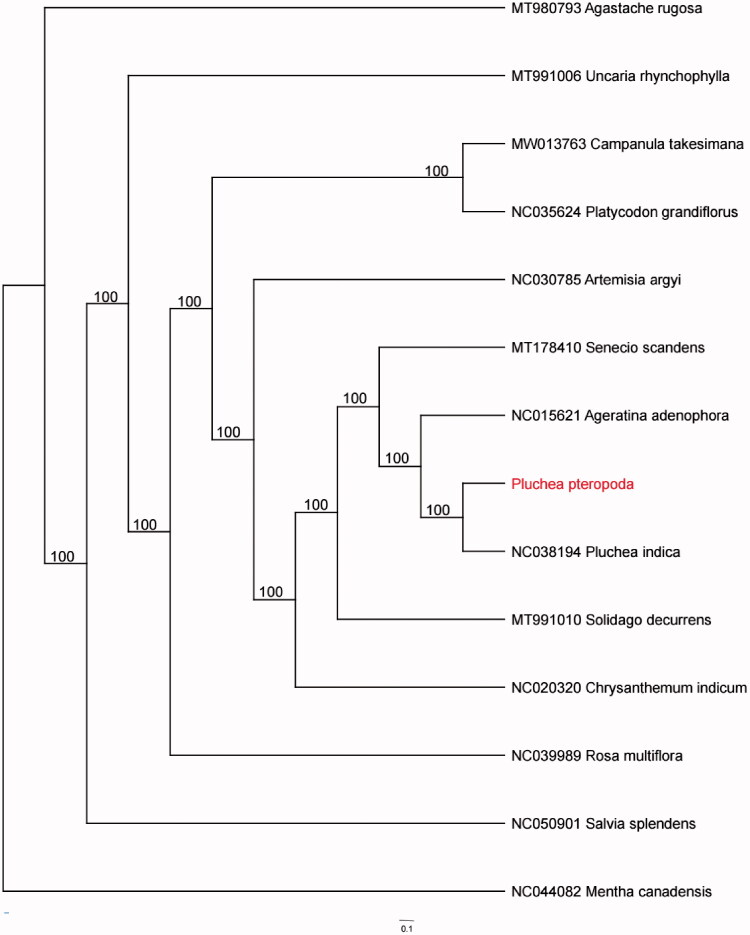
Maximum-likelihood (ML) tree based on the chloroplast genome of 14 taxa. The numbers at the nodes are bootstrap values from 1000 replicates.

## Data Availability

The data that support the findings of this study are openly available in GenBank at https://www.ncbi.nlm.nih.gov/genbank/. See supplementary material for reference numbers.
